# Comparison of networks of loneliness, depressive symptoms, and anxiety symptoms in at-risk community-dwelling older adults before and during COVID-19

**DOI:** 10.1038/s41598-024-65533-z

**Published:** 2024-06-26

**Authors:** Tianyin Liu, Yun-Han Wang, Zuna Loong Yee Ng, Wen Zhang, Stephanie Ming Yin Wong, Gloria Hoi-Yan Wong, Terry Yat-Sang Lum

**Affiliations:** 1 Department of Applied Social Sciences, The Hong Kong Polytechnic University, Hung Hom, Hong Kong, China; 2 Department of Social Work and Social Administration, The University of Hong Kong, Hong Kong, China; 3 School of Nursing and Health Studies, Hong Kong Metropolitan University, Hong Kong, China; 4 Sau Po Centre on Ageing, The University of Hong Kong, Hong Kong, China

**Keywords:** Human behaviour, Risk factors

## Abstract

Network analysis provides an innovative approach to examining symptom-to-symptom interactions in mental health, and adverse external conditions may change the network structures. This study compared the networks of common risk factors and mental health problems (loneliness, depressive symptoms, and anxiety symptoms) in community-dwelling older people before and during COVID-19. Older adults (aged ≥ 60) at risk for depression were recruited through non-governmental organizations. Loneliness, depressive symptoms and anxiety symptoms were measured using the three-item Loneliness Scale (UCLA-3), nine-item Patient Health Questionnaire (PHQ-9), and seven-item Generalized Anxiety Disorder Scale (GAD-7), respectively. Data from 2549 (before) and 3506 (during COVID-19) respondents were included using propensity score matching. *Being restless* (GAD-7-item5) was most central, indicated by Expected Influence, in both pre and during COVID-19 networks despite low severity (mean score). The network during COVID-19 had higher global strength and edge variability than the pre-pandemic network, suggesting easier symptom spread and potentially more complex symptom presentation. In addition, *feeling isolated from others* (UCLA-3-item3) had stronger connections with *feeling worthless/guilty* (PHQ-9-item6) and anticipatory anxiety (GAD-7-item7) during COVID-19 than before. These findings may enhance our knowledge of the symptom structure of common mental health problems and the impacts of the pandemic. Targeting central symptoms may offer novel preventive strategies for older people.

## Introduction

Despite COVID-19 no longer posing a global health emergency, it has adversely impacted people’s emotional well-being worldwide^1^. The pandemic hit older adults harder than the general population^2^. Before COVID-19, older adults were already facing multiple challenges, such as chronic illness, social isolation, bereavement, and barriers to accessing mental health services, including reduced mobility, lack of awareness, and stigma^3^. Therefore, common mental health problems, such as depression and anxiety, are common in older people, with depressive and anxiety symptoms being more prevalent than clinical syndromes. Systematic reviews suggested the prevalence of late-life depressive symptoms to be up to 28.4%^4^, and the prevalence of anxiety varying widely in the community (1.2–14%) and clinical (1–28%) settings for older people^5^. The pandemic has heightened feelings of isolation, fear of infection, and disruptions in routine healthcare services, further contributing to mental health challenges faced by older people^6^. These factors led to an increased prevalence of depressive and anxiety symptoms, especially among older persons with chronic illness during COVID-19, with 61% and 85% for depressive and anxiety symptoms, respectively^7^. Loneliness is another common issue experienced by older people, and the association between loneliness and mental health consequences has been consistently established^8^. For older people, the alteration of social networks, often due to retirement or the passing of a spouse, can increase loneliness and impact their quality of life and mental health^8^. Loneliness has been identified as both a risk factor for mental illness and as a mental health problem warranting attention^9^. A recent systematic review and meta-analysis during COVID-19 reported a pooled prevalence of loneliness as 28.6% (95% CI: 22.9–35.0%)^10^. During COVID-19, many disease mitigation measures, such as social distancing and lockdown, were reinforced to curb the spread of the virus and prevent at-risk groups from catching the virus. However, such measures escalated the feeling of loneliness in older adults, increasing the prevalence of mental health problems^11,12^. More targeted interventions are therefore needed to promote older persons’ mental health.

Network analysis has been utilized to understand mental health and psychiatric symptoms to develop more targeted interventions. In contrast to the traditional psychopathology theory derived from understanding physical disease, that is, the common cause theory of mental disorders^13,14^, the *network approach to psychopathology* perceives mental disorders as the result of a constellation of symptoms that interact with each other and, therefore, focuses on understanding symptom interactions^15,16^. In network analysis, each symptom is represented as a *node*, whereas the association between two nodes is shown as an *edge*. The importance of a symptom is reflected by centrality, and there are different types of centrality indices that indicate how a node exerts influence on the rest of the network^17^. Aliberti and Oliveira argue that the network approach has particular implications in advancing geriatrics or gerontology research, given that older adults’ health conditions often result from complex interactions between multimorbidity and geriatric syndromes^18^.

Mental disorders, especially common ones such as depression and anxiety, do not have conclusive and clear pathogenic pathways like physical diseases^19^. There is large symptom variability between individuals diagnosed with the same disorder^20,21^. Many factors, including age, gender and culture, may influence the presentation of symptoms^22^. Stressful events, interacting with the network but not included in it, may trigger specific symptoms and eventually spread through the whole network via the *edges*^5^, or make the symptoms more densely connected and have a higher chance of maintaining an “active state”^23^. COVID-19 was a collective stressful event, and understanding its impact on older people’s mental health from a network approach may deepen our knowledge of the effects of external factors on network structure and offer insight into targeted intervention design.

There has been a surge of studies into the mental health symptom networks during COVID-19. Several cross-sectional studies have been undertaken. For example, Cheung et al. found in the Hong Kong general population that *guilt*, *sad mood*, and *energy* symptoms were central in adults’ depression network during the COVID-19 pandemic^24^. In another study, Jin et al. identified *sad mood*, *guilt*, *motor problems*, and *lack of energy* as central symptoms in older adults’ networks in Hong Kong^25^. In a sample of older Chinese with disabilities, researchers found that *felt sadness*, *uncontrollable worry*, and *trouble relaxing* were the most central symptoms in the anxiety-depression network, which may be potential targets for intervention^26^. Hoffart et al. examined the networks of pandemic-related states (that is, loneliness, fear of infection, and financial worry), depression, and anxiety among adults in Norway^27^. They found that different pandemic-related states were associated with different anxiety and depression symptoms, and loneliness was specifically associated with various depression symptoms during the COVID-19 lockdown^27^. However, in another study examining the networks of loneliness, anxiety, and depression, Owczarek et al. found that loneliness was more distinct from anxiety and depression symptoms as reflected by low bridge expected influence (BEI) during the first lockdown in the UK^28^. In network theory, BEI expresses a node’s connectivity with the other network^29^. When applied to the understanding of symptom networks, the higher the BEI, the greater the connection between a symptom of one network (e.g., depressive symptom) with the symptom of the other network (e.g., somatic symptom clusters). Targeting central and high BEI symptoms may have ripple effects that deactivate the symptom network and, therefore, be more cost-effective.

The variations in research populations, measurement, and statistical analysis may lead to differences in study findings. Nevertheless, these studies have deepened our understanding of symptom-level interactions and may offer insights for designing targeted interventions. However, most existing studies are based on cross-sectional design; more longitudinal studies are needed to draw causal links and implement more targeted intervention strategies at different pandemic stages^28,30^.

To date, research examining the symptom networks of loneliness, anxiety, and depression among older adults remains scarce. In addition, limited studies have compared the symptom networks in older adults before and during the pandemic. Yu and Mahendran found that the associations between social isolation and affective symptoms were strengthened among older adults during the COVID-19 lockdown in Singapore^30^. However, global strength, in other words, overall network connectivity, did not significantly differ when pre-pandemic and lockdown comparisons were made^30^. Ramos-Vera et al. found that depressive symptoms and loneliness reinforced the experience of nervousness in older adults over two pandemic waves^31^, but this study could not make comparisons with pre-pandemic networks. Odenthal et al. utilized longitudinal data to examine the consistency of the network between loneliness and mental health symptoms before COVID-19 and the third incidence peak in the UK^32^. Their research suggests that the symptom networks were consistent across time, and loneliness and self-worth were identified as key symptoms to target for distress in older adults aged 50 years and over^32^. Apart from this study, however, no other study has compared the loneliness, anxiety, and depressive symptoms network in older people before and after the pandemic started, especially among at-risk groups. All in all, it remains unclear whether and how the symptom networks in older adults with mental health risks change in the presence of COVID-19 stressors.

Given the research gaps, this study aimed to: (1) investigate the networks of loneliness, depressive symptoms, and anxiety symptoms in community-dwelling older Chinese at risk of mental health problems before COVID-19, (2) during COVID-19, and (3) compare the two networks at different timepoints by propensity score matching (PSM). Several studies have used PSM previously to reduce the imbalance between cross-sectional populations recruited at different times and compared networks between them^33,34^. In the absence of longitudinal data, PSM may be a viable option to estimate the causal effects^35^. Understanding the interactions between symptoms of common mental health problems may aid professionals and policymakers in formulating cost-effective strategies to support the well-being of the ageing population.

## Methods

### Study setting and participants

This cross-sectional comparative study was conducted between October 2016 and December 2022 in Hong Kong. Data were retrieved from the baseline assessment of participants enrolled in the JoyAge project (“Jockey Club Holistic Support Project for Elderly Mental Wellness”) in Hong Kong from 2016 to 2019 (Phase I: pre-COVID-19) and 2020 to 2023 (Phase II: during COVID-19) (ClinicalTrials.gov NCT03593889 and NCT04863300, respectively)^36^. The university research team partnered with community aged care and mental health care service units, all of which were non-governmental organizations (NGOs), to provide standardized JoyAge services to community-dwelling older Chinese at risk of depression or with depressive symptoms. The JoyAge service incorporates collaborative stepped-care and peer support services and is delivered by trained social workers and peer supporters. Details of the service model, project implementation, and study protocol can be found in the published protocol and project website^36^.

All participants were recruited through JoyAge partner NGOs. Inclusion criteria were: (1) aged 60 or above; (2) having mild depressive symptoms or above (PHQ9 total scores > 4); (3) being able to provide informed consent to participate. Exclusion criteria were: (1) known history of autism, intellectual disability, schizophrenia-spectrum disorder, bipolar disorder, Parkinson’s disease, or dementia; and (2) (temporary exclusion criteria) imminent suicidal risk. We used the age of 60 as the cutoff, because in a report to the World Health Organization, the authors stated that there was no United Nations (UN) standard numerical criterion, but the UN agreed that the cutoff should be 60+ years to refer to the older population^37^. In addition, for statistical and public administrative purposes, many countries and regions, including Hong Kong, also set age 60 as eligible for citizens to receive certain benefits and welfare^38^.

Hong Kong experienced various community outbreaks of COVID-19 from early 2020 to 2023. In the first year of the COVID-19 outbreak, Hong Kong, a city with high mobility and population density, maintained a relatively low number of infections compared with nearby cities. Based on the experience of SARS in 2003, the community complied with the imposed restrictions set by the government and demonstrated good awareness in terms of maintaining personal hygiene and self-isolation. Examples of imposed restrictions included compulsory quarantine, suspended flights, mandatory confinement following positive testing, and curtailed hours to access community facilities^39^. However, while most countries started to resume social and economic activities in 2021 by “living with COVID”, a “dynamic COVID-zero” strategy was maintained in Hong Kong through strict social distancing and compulsory quarantine measures^39^. Although this approach appeared effective in the first two years, Hong Kong experienced an unexpected surge in COVID-19 cases during the emergence of the Omicron variant in early 2022^40^. The study was reviewed and approved by the Human Research Ethics Committee (HREC) of the University of Hong Kong, and all respondents gave written consent to participate in this study. The study procedures have been performed in accordance with the Declaration of Helsinki.

### Measurements

#### Loneliness

The Three-Item Loneliness Scale (UCLA-3) is adapted from the Revised UCLA Loneliness Scale to assess the subjective feeling of loneliness through three questions asking how often a person feels (i) lack of companionship, (ii) left out, and (iii) isolated^41^. A validated Chinese version was used with a 4-point Likert scale scoring from 0 (not at all) to 3 (nearly every day), giving a total score ranging between 0 and 9^42^. The internal consistency obtained by the scale was good (Cronbach’s α = 0.82) in this sample.

#### Depressive symptoms

Depressive symptoms were measured by the validated Chinese version of the Patient Health Questionnaire-9 item (PHQ-9) for older people^43^. It is a 9-item instrument that assesses the frequency of the main depressive symptoms during the past two weeks. Items are rated on a 4-point Likert scale ranging from 0 (not at all) to 3 (nearly every day). The total score can range from 0 to 27, with higher scores reflecting more severe depressive symptoms. Scores of 5–9, 10–14, 15–19, and 20 and above represent mild, moderate, moderately severe, and severe depressive symptom levels, respectively. The internal consistency of the Chinese version of PHQ-9 was acceptable in the current study (Cronbach's α = 0.62).

#### Anxiety symptoms

Anxiety symptoms were measured using the Generalized Anxiety Disorder 7-item scale (GAD-7)^44^. Responses to each anxiety symptom are rated on a 4-point Likert scale of its frequency of occurrence from 0 (not at all) to 3 (nearly every day), with total scores ranging from 0 to 21 and higher scores reflecting more severe anxiety symptoms. The internal consistency of the Chinese version of GAD-7 in this study was good (Cronbach's α = 0.88).

#### Covariates

The following covariates were measured: age (years), gender (male vs female), education level (no formal education, primary, secondary, associate and above), mobility (walking without help, walking with a stick, needing other help to move), low income (recipient of government subsidy vs non-recipient), living arrangements (living alone vs living with other(s)), and marital status (single, married/cohabiting, widowed, separated/divorced).

### Data analyses

All analyses used R (version 4.2.3).

#### Propensity score matching model

A propensity score model was developed to increase the balance between the pre-COVID-19 and the COVID-19 groups. Given the shortcomings of the linear model, particularly with skewed data, the propensity score was estimated using a probit regression^35^. The R package *MatchIt* was used for the analysis^45^, and all covariates were used for the matching.

#### Network estimation

Following previous studies, we checked the informativeness (standard deviation of the item) and redundancy (< 25% of significantly different correlations) of the items using functions from R packages *compareGroups* and *networktools*, respectively^46,47^. In the study’s network structure, each node represents one unique item from the PHQ-9, GAD-7, and UCLA-3 measures. The connections, namely, edges, between nodes represent the partial correlation between the two nodes, and the strength of edges is referred to as *weight*. The Gaussian Graphical Model (GGM) was applied to estimate the network based on partial correlation coefficients^48^. The GGM was regularized by the graphical Lasso based on an extended Bayesian information criterion (EBICglasso) with a tuning parameter (default: γ = 0.5)^48^. The purpose was to reduce false positives and to build a more parsimonious model.

#### Centrality

Central symptoms were identified via expected influence (EI), which is the summed weight of all edges extending from a given node^49^. Three other major centrality indices, namely, Betweenness, Closeness, and Strength, were also computed to examine which symptoms were most important in the loneliness–depression–anxiety symptoms network^50,51^. Strength is the absolute sum of edge weights connected to a node, Betweenness refers to the frequency of a node lying on all the shortest paths between other nodes, while Closeness is calculated as the inverse of the sum of the distance from a node to all other nodes in the network^16^. Betweenness and closeness have demonstrated poor reliability in psychopathology network studies^48^. Compared to strength, EI has been suggested to be more reliable as it considers both positive and negative edges^48^. Therefore, we reported EI centrality in the main analysis and all other centrality measures in the supplementary materials. The R packages *networktools* and *qgraph* were used to perform the analyses^47,52^.

#### Network accuracy and stability

We followed previous studies and examined the accuracy and stability of the network models. First, we used non-parametric bootstrapping to bootstrap the 95% confidence intervals (CI) of the edge weights, providing an estimate of the accuracy of edges in the networks, with narrower CIs indicating a more trustworthy network^48,53^. Second, we used the correlation stability coefficient (CS-C) to assess the stability of the centrality indices subset bootstrap, that is, by dropping participants and re-estimating the network^54^. CS-C represented the maximum proportion of cases that can be dropped (with 95% probability) to maintain a correlation larger than 0.7 with the original centrality value. Thresholds of 0.25 suggest modest stability, and 0.5 suggest strong/high metric stability^48^. The stability of EI was reported in the main analysis, and stability of other centrality measures, including Betweenness, Closeness, and Strength, were reported in Supplementary materials. The R package *bootnet* was used to perform the analyses^49^.

#### Association between symptom severity, variability, and centrality index

We calculated the associations between the centrality index (EI) and the mean score of PHQ-9, GAD-7, and UCLA-3 items using Spearman’s rank-order correlation to test whether the most central symptoms were the most severe ones^55^. We also calculated the association between the centrality index and standard deviation for all the items with Spearman’s rank-order to test whether symptom centrality could be attributed to the items’ differential variability^55^.

#### Comparison of network characteristics by time

Adapted from previous studies, differences between the networks of the two timepoints were assessed using the Network Comparison Test (NCT) in the *R* package *NetworkComparisonTest* (version 2.2.1)^56^. This test uses 1000 permutations to compare the global network strengths (absolute sum of all edge weights) and network structures (edge weight distributions) between the two networks. Finally, the differences in strength for each edge connecting two nodes were compared between the two networks after controlling for multiple tests (Holm–Bonferroni correction of *p* values).

## Results

### Participants’ characteristics before and after matching

**Figure 1:**
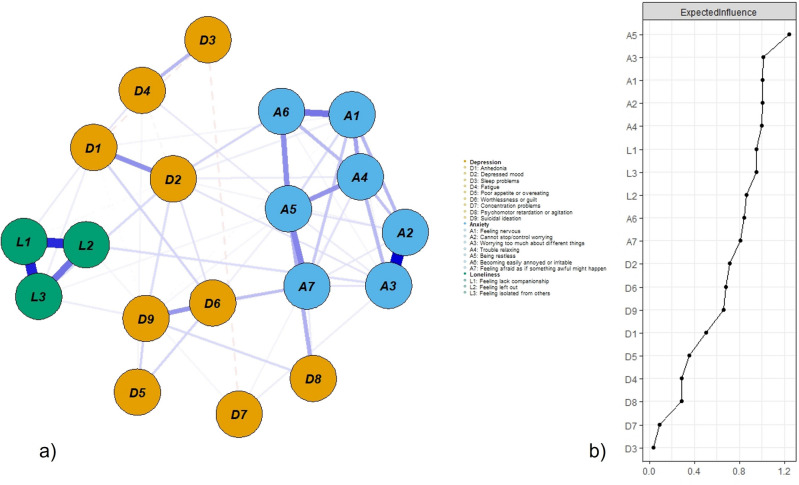
(**a**) Loneliness–depression–anxiety symptoms network before COVID-19 (N = 2549). Green circles represent loneliness items, yellow depressive symptoms, and blue anxiety symptoms. Solid edges represent positive edge weights (a positive relationship between symptoms), dashed edges represent negative edge weights (a negative relationship between symptoms), and the thickness of the edges represents the strength of associations between nodes. (**b**) Standardized centrality indices (Expected Influence) of the network structure of loneliness–depression–anxiety symptoms (*Z *scores).

In total, 3150 participants (*M*_age_ = 75.61 years, *SD* = 10.57) were enrolled pre-COVID-19 (from January 2016 to December 2019), and 4514 (*M*_age_ = 73.53 years, *SD* = 8.00) during COVID-19 (from January 2020 to January 2023). They differed in all socio-demographic variables except for living arrangements and marital status. After propensity score matching using a *probit* regression method on covariates, 2549 (*M*_age_ = 73.53 years, *SD* = 8.95) and 3506 (*M*_age_ = 73.69 years, *SD* = 7.98) older adults were included in the pre-COVID-19 and during COVID-19 groups, respectively. All standardized mean differences for the covariates were below 0.1 (Fig. 1, Supplementary Table 1). Table 1 summarizes participants’ basic socio-demographic characteristics and mental health measures before and after matching. Participants recruited during COVID-19 scored significantly higher than those recruited before COVID-19 on PHQ-9 (*t* (6053) = 23.08, *p* < 0.001), GAD-7 (*t* (6053) = 17.04, *p* < 0.001), and UCLA-3 (*t* (6053) = 5.67, *p* < 0.001) after matching.

**Table 1:** Demographic characteristics of participants recruited before (2016–2019) and during COVID-19 (2020–2023) before and after propensity score matching.

Mean (SD)/N (%)	Before matching			After matching		
	Pre-COVID-19 (N = 3150 with missing)	During COVID-19 (N = 4514 with missing)	*p*	Pre-COVID-19 (N = 2549)	During COVID-19 (N = 3506)	*p*
Age, years	75.61 (10.57)	73.53 (8.00)	< 0.001	73.57 (8.41)	73.69 (8.00)	0.07
Gender, female	2229 (77.7%)	3753 (83.6%)	< 0.001	1988 (78.0%)	2805 (80.0%)	0.06
Education level			< 0.001			< 0.001
No formal education	1003 (34.5%)	772 (17.2%)		876 (34.4%)	602 (17.2%)	
Primary school	1099 (37.8%)	1850 (41.0%)		947 (37.2%)	1445 (41.2%)	
Secondary school	707 (24.2%)	1594 (35.3%)		639 (25.1%)	1236 (35.3%)	
Associate degree and above	102 (3.5%)	287 (6.4%)		87 (3.4%)	223 (6.3%)	
Mobility			< 0.05			0.18
Able to walk without any help	1801 (61.8%)	3250 (74.6%)		1565 (61.4%)	2233 (63.7%)	
Able to walk with stick	972 (33.4%)	985 (22.6%)		861 (33.8%)	1174 (33.5%)	
Reliant on other help for mobility	139 (4.8%)	122 (2.8%)		123 (4.8%)	99 (2.8%)	
Low-income, recipient of government subsidy	938 (31.2%)	853 (19.0%)	< 0.001	762 (29.9%)	765 (21.8%)	< 0.001
Living status, living alone	1247 (41.5%)	1790 (39.9%)	0.17	1034 (40.6%)	1430 (40.8%)	0.26
Marital status			0.19			0.39
Single	176 (5.8%)	303 (6.7%)		155 (6.1%)	246 (7.0%)	
Married/cohabiting	1167 (39.6%)	1778 (41.2%)		1037 (40.4%)	1338 (38.1%)	
Widowed	1311 (44.5%)	1612 (37.3%)		1120 (43.9%)	1477 (42.1%)	
Separated/divorced	292 (10%)	627 (14.5%)		237 (9.3%)	445 (12.7%)	
Mental health measures						
Depression, PHQ-9 total scores	8.43 (3.52)	9.98 (3.73)	< 0.001	8.17 (3.28)	10.31 (3.74)	< 0.001
Anxiety, GAD-7 total scores	5.64 (4.67)	7.14 (4.6)	< 0.001	5.59 (4.62)	7.61 (4.67)	< 0.001
Loneliness, UCLA-3 total scores	4.57 (2.9)	4.92 (2.27)	< 0.001	4.53 (2.89)	4.90 (2.28)	< 0.001

*GAD-7* the Generalized Anxiety Disorder 7-item scale, *PHQ-9* the Patient Health Questionnaire-9 item scale, *SD* standard deviation, *UCLA-3* Three-Item Loneliness Scale.

All individual items were above the informativeness threshold (that is ± 2.5 *SD* below the mean level), and no item was considered statistically redundant in the loneliness–depression–anxiety symptoms network before COVID-19. GAD-7 items (hereafter A) A1 (*feeling nervous*) and A7 (*feeling afraid as if something awful might happen*) had less than 25% of significantly different correlations from others. However, we included all clinical measure items to keep the two network nodes consistent. Supplementary Table 2 summarizes all individual items and comparisons between the two times. Participants during COVID-19 reported higher mean severity ratings in all items than pre-COVID-19 participants.

### Model 1: Loneliness–depression–anxiety symptoms network before COVID-19

**Figure 2:**
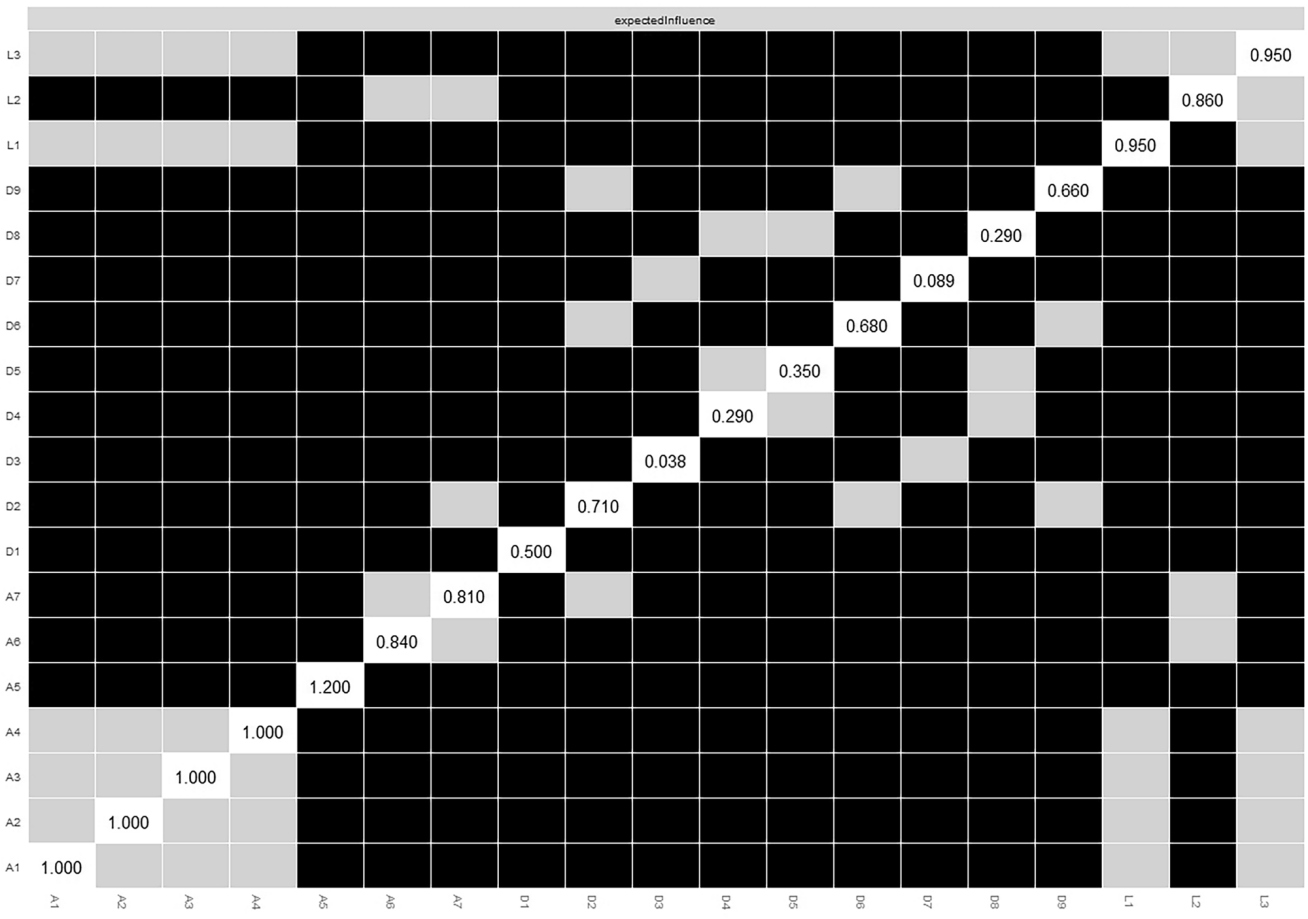
Bootstrapped difference tests between node expected influence in the pre-COVID-19 network. Gray boxes indicate nodes that do not significantly differ from one another. Black boxes represent nodes that differ significantly from one another (α = 0.05). White boxes show the values of node expected influence.

Figure 1a displays the network structure of the pre-COVID-19 loneliness–depression–anxiety symptoms network. There were 95 non-zero edges out of 171 edges based on 19 nodes (symptoms), and the mean weight of all edges was 0.039. The largest edge weights in the network were between A2 (*unable to stop or control worrying*) and A3 (*worrying too much about different things*) (part *r* = 0.50), and between UCLA-3 items (hereafter L) L2 (*feeling left out*) and L3 (*feeling isolated from others*) (part *r* = 0.44). The centrality index Expected Influence (EI) shown in Fig. 1b indicates that A5 (*being restless*) was the most central symptom, followed by A3 (*worrying too much about different things*). The bootstrapped results of EI also confirmed the most central role of A5, but there was no difference between A1, A2, A3, and A4 (Fig. 2). Supplementary Fig. 2a summarizes the results from other centrality indices (Strength, Closeness, Betweenness). In general, in terms of EI, anxiety symptoms were most central in this network, followed by loneliness, and depressive symptoms were less central, especially the somatic (PHQ-9 items, hereafter D) D3 (*sleep problems*) and cognitive-motor D7 (*concentration problems*) symptoms.

These results are interesting because in terms of severity rating (that is, frequency of experiencing the symptoms, synchronized in three scales), participants scored highest in L3 (*feeling isolated from others*; *mean* = 1.69, *SD* = 1.10) and D3 (*sleep problems*; *mean* = 1.68, *SD* = 0.97); while A5 received an average rating of 0.69 (*SD* = 0.82), and all GAD-7 items had an average score lower than 1 (*mean* = 0.80, *SD* = 0.85), indicating those symptoms were experienced less frequently than several days in the previous two weeks. In terms of cross-scale connection, L3 had the strongest connection with D9 (*suicidal ideation*; part *r* = 0.08), which warrants attention for more targeted intervention because of the high risk indicated by D9. However, despite high mean severity, D3 (*sleep problems*) had a very weak EI on other mental health symptoms except for D4 (*fatigue*), another somatic symptom commonly reported by older people. Bivariate analysis between symptom severity (mean score) and EI revealed that they were not related (*r*_*s*_ = − 0.23, *p* = 0.34), symptom severity variance (standard deviation) was not correlated with EI either (*r*_*s*_ = − 0.05, *p* = 0.83), suggesting that symptom centrality was not related to its severity level and variability.

Regarding network stability, the case-dropping procedure showed that the EI (CS-C = 0.75) value remained stable after dropping different proportions of the sample; the EI value was the same for Strength (CS-C = 0.75), and Closeness (CS-C = 0.75), and modest for Betweenness (CS-C = 0.44) (Supplementary Fig. 2b). The edge weight bootstrapped difference test revealed that many comparisons among edge weights were statistically significant (Supplementary Fig. 3a), and there was substantial overlap among the 95% CIs of edge weights (Supplementary Fig. 3b).

### Model 2: Loneliness–depression–anxiety symptoms network during COVID-19

**Figure 3:**
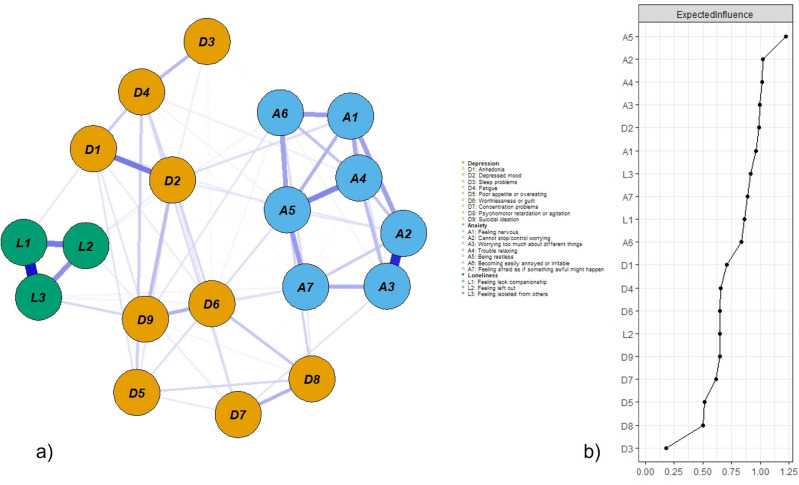
(**a**) Loneliness–depression–anxiety symptoms network during COVID-19 (N = 3506). Green circles represent loneliness items, yellow depressive symptoms, and blue anxiety symptoms. Solid edges represent positive edge weights (a positive relationship between symptoms), dashed edges represent negative edge weights (a negative relationship between symptoms), and the thickness of the edges represents the strength of associations between nodes. (**b**) Standardized centrality indices (Expected Influence) of the network structure of loneliness–depression–anxiety symptoms (*Z* scores).

**Figure 4:**
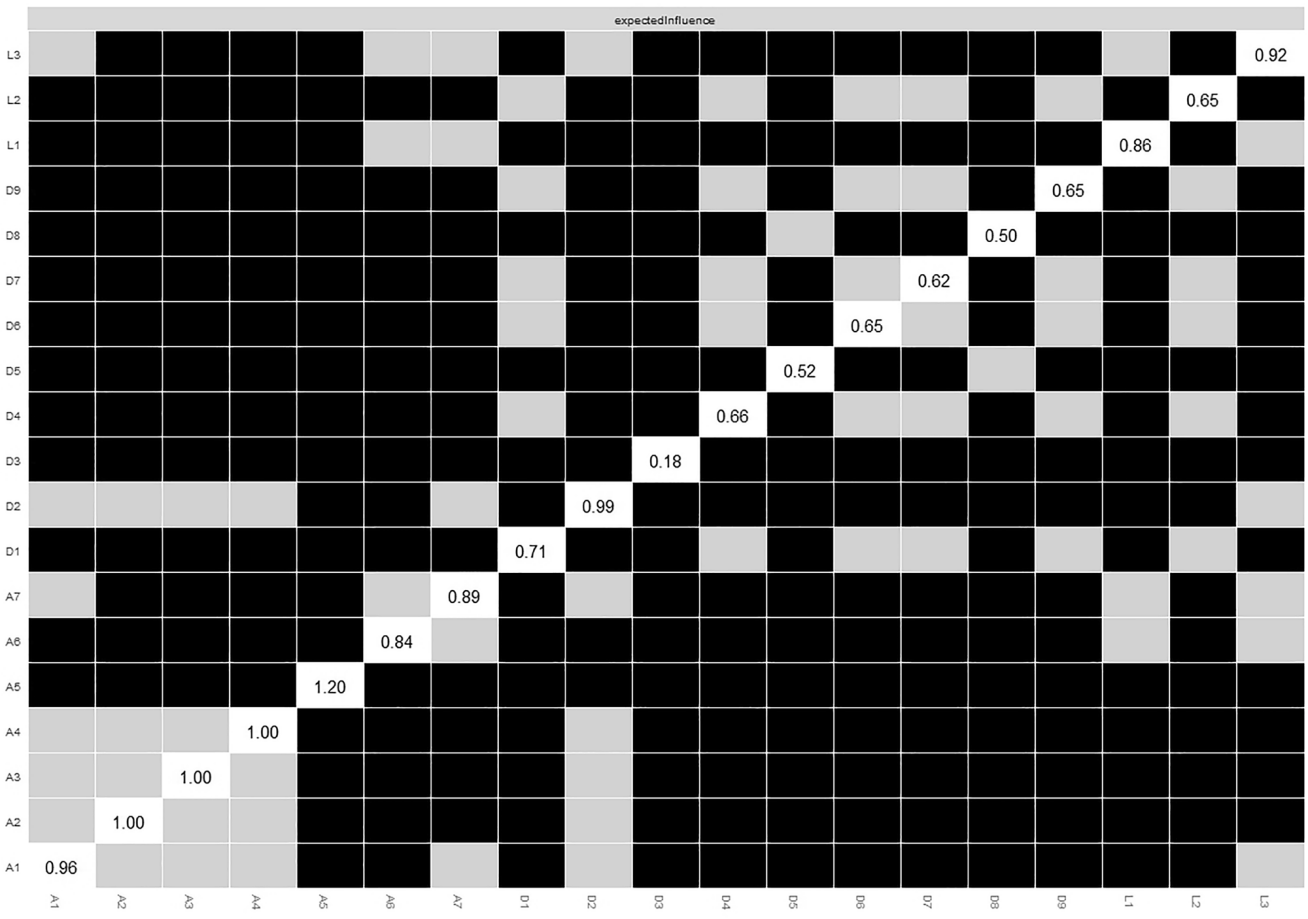
Bootstrapped difference tests between nodes expected influence in the network during COVID-19. Gray boxes indicate nodes that do not significantly differ from one another. Black boxes represent nodes that differ significantly from one another (α = 0.05). White boxes show the values of node expected influence.

Figure 3a displays the during COVID-19 network structure of the loneliness–depression–anxiety symptoms network. There were 112 non-zero edges out of 171 edges based on 19 nodes, and the mean weight of all edges was 0.043. The largest edge weights in the network were between L1 (*feeling a lack of companionship*) and L3 (*feeling isolated from others*) (part* r* = 0.47) and between A2 (*cannot stop or control worrying*) and A3 (*worrying too much about different things*) (part* r* = 0.43). The centrality index EI shown in Fig. 3b was similar to the pre-COVID-19 network, indicating that A5 (*being restless*) was the most central symptom followed by A2 (*cannot stop or control worrying*). The main difference was the increasingly more important role of D2 (*depressed mood*). The bootstrapped results of EI also confirmed the most central role of A5 and a more central role of D2, but there was no difference between A2, A3, and A4 (Fig. 4). Supplementary Fig. 4a summarizes the results from other centrality indices (Strength, Closeness, Betweenness). Similar to the pre-COVID-19 network, in terms of EI, anxiety symptoms were most central in this network, followed by loneliness; depressive symptoms were less central, except for the two cardinal depressive symptoms, namely, D2 (*depressed mood*) and D1 (*anhedonia*). The somatic D3 (*sleep problems*) and cognitive-motor D8 (*psychomotor retardation or agitation*) had the lowest centrality scores.

These results found in Model 2 (during COVID-19) are largely similar to those in Model 1 (pre-COVID-19). Anxiety symptoms had the highest centrality despite a low severity rating (*mean* A5 = 1.03, *SD* = 0.84; *mean* A2 = 1.17, *SD* = 0.87). In terms of cross-scale connection, L3 consistently has the strongest connection with D9 (*suicidal ideation*; part *r* = 0.09); at the same time, L3 also received a high severity rating (*mean* = 1.78, *SD* = 0.90), which is alarming and suggests that *feeling of isolation* deserves more attention. Similarly, despite high mean severity, D3 (*sleep problems,* mean = 1.82, SD = 0.92) had weak connections with other mental health symptoms. In the COVID-19 network, D2 (*depressed mood*) took up a more central role and had a high connection with D9 (*suicidal ideation*; part *r* = 0.13) than in the pre-COVID-19 network. Bivariate analysis between symptom severity (mean score) and EI revealed that they were not related (*r*_*s*_ = − 0.35, *p* = 0.41), symptom severity variance (standard deviation) was not correlated with strength either (*r*_*s*_ = − 0.38, *p* = 0.61), suggesting that symptom centrality was not related to its severity level and variability.

Regarding network stability, the case-dropping procedure showed that the EI (CS-C = 0.75) value remained stable after dropping different proportions of the sample. The EI value was the same for Strength (CS-C = 0.75), Closeness (CS-C = 0.75), but low for Betweenness (CS-C = 0.13) (Supplementary Fig. 4b). The edge weight bootstrapped difference test revealed that many comparisons among edge weights were statistically significant (Supplementary Fig. 5a), and there was substantial overlap among the 95% CIs of edge weights (Supplementary Fig. 5b).

### Network comparisons between the two timepoints

**Figure 5:**
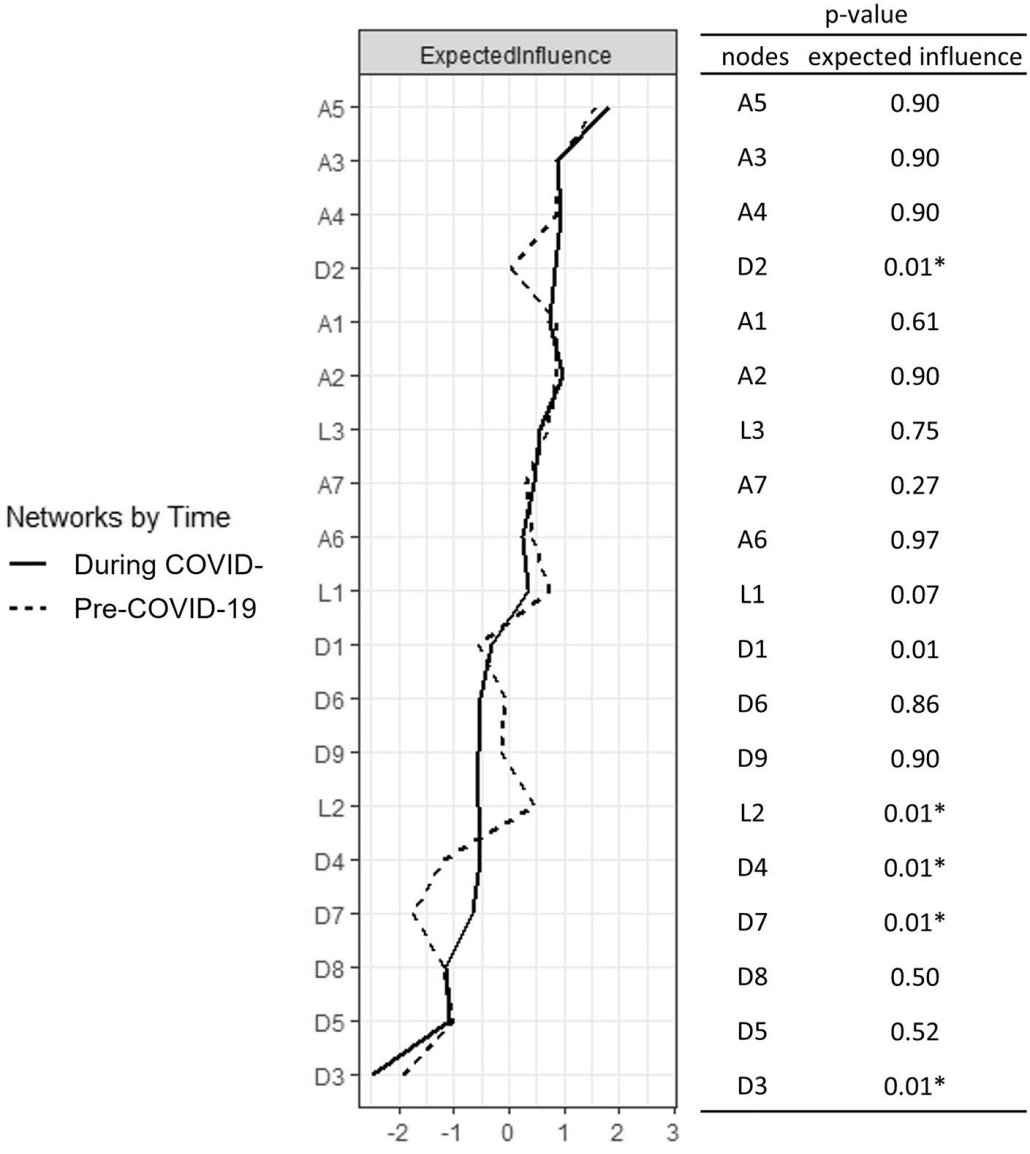
Comparison of network centrality indices between respondents recruited before and during COVID-19. *A* Anxiety symptoms, items from the Generalized Anxiety Disorder-7 item scale, *D* Depression symptoms, items from the Patient Health Questionnaire-9 item scale, *L* Loneliness, items from the UCLA-3 item loneliness scale. *p < 0.05.

We tested whether global strength, structure, and single edges differed by time. From the network comparison test (NCT) analyses, we observed that the pre-COVID-19 network and the COVID-19 network significantly differed in global strength (before = 7.13, during = 7.64; *S* = 0.51, *p* < 0.01) and distribution of edge weights (*M* = 0.160, *p* < 0.001). We also compared the centrality differences of each node, and Fig. 5 summarizes the results. Notably, D2 (*depressed mood*), D4 (*fatigue*), and D7 (*concentration problem*) had higher centrality during the COVID-19 network than before COVID-19 (*p* < 0.05).

Out of 171 edges between 19 symptoms, 19 edges had significant differences between the two networks, as summarized in Supplementary Table 4 (*p* < 0.05 after Holm–Bonferroni corrections). Among the 19 significantly different edges, 13 were within the same scales (nine depression, three anxiety, one loneliness), and six were across scales (three between anxiety and depression, two between depression and loneliness, and one between anxiety and loneliness). In general, depressive symptoms were more strongly coupled during COVID-19. One notable change was a stronger connection between L3 (*feeling isolated from others*) and D6 (*worthlessness or guilt*) and between L3 and A7 (*feeling afraid as if something awful might happen*), suggesting that during COVID-19, feeling isolated from others, possibly induced by disease mitigation measures, had a higher probability of co-occurring with *feeling worthless or guilty*, and *being afraid that something awful might happen*.

## Discussion

This study had three aims: (1) to investigate the networks of loneliness, depression, and anxiety symptoms in community-dwelling older Chinese at risk of mental health problems before and (2) after COVID-19, and (3) to compare the two networks. To our knowledge, this is the first study to characterize the networks of the aforementioned common mental health problems in older Chinese and compare the pre-COVID-19 and COVID-19 networks using a propensity score matching approach. Loneliness, depressive symptoms, and anxiety symptoms are common in older people, and a deeper understanding of the symptom structures may inform mental health professionals in developing more targeted interventions. In addition, some of the findings may be relevant for policymakers in balancing disease mitigation measures and older people’s mental health needs in future public health crises.

First, we found that in the loneliness–depression–anxiety symptoms network in community-dwelling older people at risk of depression, A5 (*being restless*) was the most central symptom. It is arguably an anxiety symptom that has some conceptual overlap with depression, and it was most central in both networks regardless of time, suggesting a relatively robust central role. Its centrality was not associated with symptom severity or symptom variability. In terms of symptom severity, this community sample reported a higher frequency of experiencing loneliness, followed by depressive symptoms, but least frequent anxiety. This disassociation between symptom severity and centrality was consistent with findings from previous studies. For example, Fried demonstrated that insomnia exerted a considerable influence on other symptoms and had higher centrality despite comparatively lesser severity within the context of major depressive disorder (MDD)^20^. Beard reported in a network analysis of depression and anxiety symptoms in a psychiatric sample that symptoms like *feeling afraid that something awful might happen* and *feeling easily annoyed or irritated* were the most central but not always the most severe^57^. These findings suggest that the most central symptoms in a network might not necessarily be the most severe, but they could play a significant role due to their diverse or strong connections with other symptoms, therefore, effectively activating the problematic network. These central but less severe symptoms might be easily overlooked in the traditional psychopathology framework, especially if professionals simply use sum scores to differentiate mental health needs. More research is needed to gain a clearer understanding of symptom networks and disassociation between centrality and severity.

Second, *being restless* and *feeling isolated from others* in older adults need more attention. Research has shown that older adults can experience anxiety symptoms somewhat differently from younger people. For instance, some older adults might display more physical symptoms of anxiety, such as *restlessness* or *an inability to relax*, rather than cognitive symptoms often associated with anxiety, like pervasive worry^58^. Even if r*estlessness* receives a high severity rating in older adults, it could be attributed to other comorbidities like chronic pain and overlooked as a mental health symptom^59^. Hence, the presence or absence of symptoms such as restlessness could help discern the level of anxiety in older adults. Our findings augment the literature by suggesting its central role in driving the loneliness–depression–anxiety symptoms network in community-dwelling older adults. Although not among the most central symptoms in this study, *feeling isolated from others* had significant connections with *suicidal ideation* in networks other than the depressive symptom network. During COVID-19, older people experienced elevated social isolation and loneliness, which may be attributed to disease mitigation measures such as social distancing^60^. Some studies argue that loneliness, but not social distancing, was associated with suicidal ideation during the COVID-19 pandemic^61^. Researchers have suggested promoting social connection as public health messaging, mobilizing diverse social support networks, including the family and community, and developing technology-based interventions to increase accessibility to reduce loneliness^60^. Our findings also support interventions targeting loneliness, which could have a ripple effect on reduced suicidal risk. For example, community aged care services can include screening for loneliness using the UCLA-3item scale. For those who screened positive, particularly on the third item, *feeling isolated from others,* targeted interventions to reappraisal the loneliness and self-harm thoughts using cognitive behavioral therapy (CBT), to increase social engagement and peer support such as community activities and volunteer opportunities, or to increase access to services such as telehealth during times of public health crisis may be offered to older people^62–64^.

Third, compared to the pre-COVID-19 network, the loneliness–depression–anxiety symptoms network during COVID-19 had significantly larger global strength and distribution of edge weights, which may reflect the influence of the pandemic on wider symptom activation and more complex presentation of symptom associations. The higher global strength of the network suggests that symptoms may have a greater impact on each other, and older people may experience more persistent symptoms during COVID-19, as the activation of one symptom could lead to a higher likelihood of symptoms reinforcing one another. This finding differs from that of Yu and Mahendran, who reported no significant difference in the global strength of pre-COVID-19 and lockdown networks despite finding altered dynamics between symptoms brought by lockdown^30^. Their study collected data from mainly healthy community-dwelling older people with a pre-COVID-19 mean score on the Geriatric Depression Scale (GDS) of 1.02 and a lockdown GDS mean score of 2.11, indicating no depression risk^30^. In contrast, our sample comprised those at risk of depression, with a mean PHQ-9 score exceeding 4. We speculate that the variation in results could be attributed to having different respondent characteristics. It is plausible that those without mental health risks pre-COVID-19 may have higher resilience and buffer when facing stressful events like lockdown, while changes in the external environment are more likely to cause “cascading failure” among those already at-risk^65^. Our findings may echo previous research in drawing attention to those with pre-existing mental health conditions^66^, adding evidence from a network perspective of psychopathology.

Four, compared to the pre-COVID-19 network, the depressive symptoms (D2—*depressed mood*, D4—*fatigue*, and D7—*concentration problem*) showed higher centrality, and two edges connecting symptoms from different scales, L3 (*feeling isolated from others*) and D6 (*feelings of worthlessness or guilt*), and L3 and A7 (*feeling afraid as if something awful might happen*) became stronger during COVID-19. We did not specify domains of symptoms in the network analysis (different initials just for naming the variables). Also, we did not examine the bridge expected influence because, conceptually, some depression and anxiety symptoms may overlap, as in the case of depression and loneliness. Nevertheless, the results showed that *feeling isolated from others* was more likely to couple with *feelings of worthlessness or guilt*, a significant depressive symptom found to be associated with the presence of psychotic features and relapse of depression^67,68^, and more likely to co-occur with *feeling afraid as if something awful might happen*, a symptom that indicates anticipatory anxiety and has a strong connection with other anxiety symptoms within the network^69^. These findings emphasize the importance of targeting loneliness to reduce coactivation between symptoms. Finally, compared to pre-COVID-19, all loneliness, depression, and anxiety symptoms received higher severity ratings. Despite not being a longitudinal study, through PSM, the two samples were largely matched, and the elevated mental health risks are consistent with other studies^2^.

This study had several strengths. First, it used a network approach to examine common mental health symptoms, which can capture the complexity, interconnectedness, and dynamic nature of mental health networks. With more conventional research methods, the focus would be on more severe symptoms and less on the function of individual symptoms in maintaining the whole network or linking to other symptom clusters. Second, data from this study were collected from community-dwelling older people at risk of depression, who were rated not only on depressive symptoms but also anxiety and loneliness, two other common comorbid mental health problems in old age. Given that comorbidity is more common than not, this sample provides a more realistic depiction of mental health challenges experienced by community-dwelling older people than other studies focusing on one or two conditions. Third, this study used PSM to minimize the imbalance between participants recruited before and during the COVID-19 community breakout and offered a different perspective on examining the mental health impact of the pandemic, in other words, the changes in symptom network structure. Finally, data from this study were retrieved from an ongoing large-scale service project; participants may not typically participate in research or were previously unknown to mental health services. Therefore, the study’s results may have direct service implications for mental health providers to design targeted interventions and examine their impact on the symptom network structure through longitudinal studies.

Several limitations need to be considered in interpreting the results of this study. First, the edges in each network were calculated with cross-sectional data, precluding estimations of important network characteristics such as the direction of edges. Although PSM may offer some insight into COVID-19’s influence on the network, the data were nevertheless retrieved from different individuals. Experimental or prospective designs are required to test the assumptions underlying causality^57^. Second, although the *qgraph glasso* and EBIC procedure conducts model comparison that maximizes fit, there are no clear best good-of-fit metrics for network comparison tests^70^. Third, although all items were from standardized measurements, some items may have conceptual overlaps (for example, A2 *cannot stop/control worrying*, and A3 *worrying too much about different things*) that are especially harder for community-dwelling older people to differentiate, and potentially artificially inflating edge weights and centrality, in particular among anxiety symptoms.

In summary, the results of the study reveal the importance of a specific anxiety symptom, *restlessness*, and loneliness item, *feeling isolated from others*, in the network of loneliness–depression–anxiety symptoms among community-dwelling older people at risk of depression, and their centrality was disassociated from symptom severity or variability. Comparing networks at two timepoints, we found that the network during COVID-19 had larger global strength and edge variability, suggesting wider symptom activation and more complex presentation of symptom associations. In particular, *depressed mood* became more important in connecting with other symptoms in the network, and *feeling isolated from others* was more likely to co-occur with important depression and anxiety symptoms associated with higher relapse of depression and anticipatory anxiety. These findings may assist mental health service providers to make more personalized interventions for older people and inform policymakers to balance disease mitigation measures and people’s mental health needs.

### Supplementary Information

Supplementary Information.

## Data Availability

The data, analytical codes, and materials that support the findings of this study are available from the corresponding author upon reasonable request.
