# Supplementary material for: Comparison of networks of loneliness, depressive symptoms, and anxiety symptoms in at-risk community-dwelling older adults before and during COVID-19
Source: Sci Rep. 2024 Jun 26;14:14737. doi: 10.1038/s41598-024-65533-z (PMC11208589; doi:10.1038/s41598-024-65533-z)
Supplement: Supplementary file 1 — Supplementary Information. [file 41598_2024_65533_MOESM1_ESM.docx]

**Supplementary Table 1.** Summary of balance for matched data

|  | Pre-COVID-19 means | During COVID-19 means | Std. mean difference | Variance ratio | eCDF mean | eCDF max | Std. Pair distance |
| --- | --- | --- | --- | --- | --- | --- | --- |
| Distance | 0.61 | 0.61 | 0.00 | 1.00 | 0.00 | 0.00 | 0.00 |
| Age | 73.57 | 73.49 | 0.01 | 1.03 | 0.01 | 0.02 | 0.48 |
| Gender | 0.17 | 0.16 | 0.02 | . | 0.01 | 0.01 | 0.40 |
| Education level | 2.43 | 2.39 | 0.03 | 1.05 | 0.01 | 0.02 | 0.35 |
| Mobility | 1.31 | 1.29 | 0.02 | 1.11 | 0.00 | 0.01 | 0.50 |
| Low-income | 0.21 | 0.19 | 0.04 | . | 0.02 | 0.02 | 0.39 |
| Living alone | 0.44 | 0.44 | 0.01 | . | 0.00 | 0.00 | 0.30 |
| Marital status | 2.33 | 2.24 | 0.05 | 1.08 | 0.02 | 0.03 | 0.59 |

eCDF: empirical cumulative distribution functions; Std: standardized

**Supplementary Table 2.** Means, standard deviations, *t* test, *p value*, and Cohen’s *d* in matched pre-COVID-19 (N=2549) and during COVID-19 (N=3506) groups

|  | Pre-COVID-19 | During COVID-19 | **Comparison** | |
| --- | --- | --- | --- | --- |
| **Network items** | **Mean (SD)** | **Mean (SD)** | **p** | **Cohen’s d** |
| PHQ-9_1 (D1) | 1.14 (0.93) | 1.51 (0.85) | <0.001 | 0.42 |
| PHQ-9_2 (D2) | 1.38 (0.86) | 1.61 (0.76) | <0.001 | 0.28 |
| PHQ-9_3 (D3) | **1.68 (0.97)** | **1.82 (0.92)** | <0.001 | 0.15 |
| PHQ-9_4 (D4) | 1.47 (0.88) | 1.57 (0.83) | <0.001 | 0.12 |
| PHQ-9_5 (D5) | 0.34 (0.69) | 0.73 (0.84) | <0.001 | 0.51 |
| PHQ-9_6 (D6) | 0.69 (0.88) | 1.00 (0.9) | <0.001 | 0.35 |
| PHQ-9_7 (D7) | 0.84 (0.8) | 1.08 (0.83) | <0.001 | 0.29 |
| PHQ-9_8 (D8) | 0.33 (0.62) | 0.63 (0.78) | <0.001 | 0.43 |
| PHQ-9_9 (D9) | 0.29 (0.59) | 0.36 (0.65) | <0.001 | 0.11 |
| PHQ-9 item average | 0.91 (0.80) | 1.15 (0.82) | <0.001 | 0.30 |
| GAD-7_1 (A1) | 0.93 (0.88) | 1.29 (0.84) | <0.001 | 0.42 |
| GAD-7_2 (A2) | 0.89 (0.87) | 1.17 (0.87) | <0.001 | 0.32 |
| GAD-7_3 (A3) | 0.81 (0.84) | 1.06 (0.88) | <0.001 | 0.29 |
| GAD-7_4 (A4) | 0.81 (0.89) | 1.25 (0.87) | <0.001 | 0.50 |
| GAD-7_5 (A5) | 0.69 (0.82) | 1.03 (0.84) | <0.001 | 0.41 |
| GAD-7_6 (A6) | 0.87 (0.85) | 1.03 (0.84) | <0.001 | 0.19 |
| GAD-7_7 (A7) | 0.60 (0.81) | 0.80 (0.86) | <0.001 | 0.24 |
| GAD-7 item average | 0.80 (0.85) | 1.09 (0.86) | <0.001 | 0.34 |
| UCLA-3_1 (L1) | 1.49 (1.08) | 1.69 (0.92) | <0.001 | 0.20 |
| UCLA-3_2 (L2) | 1.35 (1.06) | 1.43 (0.96) | 0.002 | 0.08 |
| UCLA-3_3 (L3) | 1.69 (1.10) | 1.78 (0.90) | <0.001 | 0.09 |
| UCLA-3 item average | 1.51 (1.08) | 1.63 (0.93) | <0.001 | 0.12 |

GAD-7: the Generalized Anxiety Disorder 7-item scale; PHQ-9: the Patient Health Questionnaire-9 item scale; SD: standard deviation; UCLA-3: Three-Item Loneliness Scale.

**Supplementary Table 3.** Adjacency matrix – left lower panel (pre-COVID-19), right upper panel (during COVID-19)

|  | D1 | D2 | D3 | D4 | D5 | D6 | D7 | D8 | D9 | A1 | A2 | A3 | A4 | A5 | A6 | A7 | L1 | L2 | L3 |
| --- | --- | --- | --- | --- | --- | --- | --- | --- | --- | --- | --- | --- | --- | --- | --- | --- | --- | --- | --- |
| D1 | - | **0.264** | 0.000 | 0.101 | 0.068 | 0.049 | 0.019 | 0.000 | 0.063 | 0.020 | 0.000 | 0.011 | 0.000 | 0.052 | 0.000 | 0.000 | 0.059 | 0.000 | 0.000 |
| D2 | **0.219** | - | 0.044 | 0.084 | 0.058 | 0.084 | 0.059 | 0.000 | 0.131 | 0.074 | 0.061 | 0.000 | 0.032 | 0.005 | 0.031 | 0.000 | 0.000 | 0.044 | 0.018 |
| D3 | -0.06 | 0.000 | - | 0.139 | 0.000 | -0.04 | 0.007 | -0.03 | 0.000 | 0.026 | 0.000 | 0.000 | 0.031 | 0.000 | 0.011 | 0.013 | 0.000 | -0.02 | 0.000 |
| D4 | 0.068 | -0.04 | 0.144 | - | 0.100 | 0.000 | 0.075 | 0.000 | 0.010 | 0.017 | 0.000 | 0.000 | 0.044 | 0.042 | 0.005 | 0.034 | 0.000 | 0.000 | 0.007 |
| D5 | 0.028 | 0.000 | 0.000 | 0.039 | - | 0.046 | 0.058 | 0.086 | 0.022 | 0.000 | 0.000 | 0.017 | 0.000 | 0.071 | 0.000 | 0.000 | 0.000 | 0.000 | -0.01 |
| D6 | 0.086 | 0.070 | 0.015 | 0.000 | 0.086 | - | 0.028 | 0.115 | 0.154 | 0.000 | 0.000 | 0.009 | 0.000 | 0.058 | 0.040 | 0.067 | 0.000 | 0.000 | 0.041 |
| D7 | 0.000 | 0.000 | -0.06 | 0.000 | 0.013 | 0.000 | - | 0.154 | 0.000 | 0.017 | 0.001 | 0.068 | 0.032 | 0.008 | 0.015 | 0.020 | 0.000 | 0.047 | 0.010 |
| D8 | -0.01 | -0.01 | -0.03 | 0.012 | 0.025 | 0.026 | 0.000 | - | 0.035 | 0.000 | 0.000 | 0.000 | 0.000 | 0.097 | 0.000 | 0.054 | 0.000 | -0.01 | 0.000 |
| D9 | 0.032 | 0.074 | 0.000 | 0.000 | 0.095 | 0.206 | 0.000 | 0.078 | - | 0.000 | 0.002 | 0.000 | 0.021 | 0.000 | 0.066 | 0.049 | 0.005 | 0.000 | 0.088 |
| A1 | 0.041 | 0.045 | 0.009 | 0.016 | 0.011 | 0.000 | 0.000 | 0.000 | 0.032 | - | 0.187 | 0.070 | 0.172 | 0.155 | 0.193 | 0.028 | 0.000 | 0.000 | 0.000 |
| A2 | 0.000 | 0.041 | 0.000 | 0.000 | 0.000 | 0.000 | 0.005 | 0.000 | 0.000 | 0.147 | - | **0.429** | 0.123 | 0.061 | 0.000 | 0.104 | 0.013 | 0.026 | 0.016 |
| A3 | 0.000 | 0.067 | 0.000 | 0.000 | 0.000 | 0.000 | 0.045 | -0.03 | 0.000 | 0.042 | **0.503** | - | 0.131 | 0.016 | 0.039 | 0.162 | 0.012 | 0.034 | 0.000 |
| A4 | 0.000 | 0.055 | 0.000 | 0.000 | 0.012 | 0.023 | 0.042 | 0.000 | 0.004 | 0.183 | 0.127 | 0.141 | - | **0.241** | 0.123 | 0.034 | 0.010 | 0.013 | 0.006 |
| A5 | 0.006 | 0.000 | 0.014 | 0.058 | 0.011 | 0.043 | 0.000 | 0.158 | 0.036 | 0.077 | 0.068 | 0.052 | **0.211** | - | 0.186 | **0.201** | 0.031 | 0.000 | 0.000 |
| A6 | 0.000 | 0.087 | 0.000 | 0.000 | 0.000 | 0.000 | 0.004 | 0.000 | 0.006 | **0.272** | 0.032 | 0.037 | 0.138 | **0.222** | - | 0.079 | 0.000 | 0.038 | 0.013 |
| A7 | 0.000 | -0.01 | 0.000 | 0.005 | 0.024 | 0.126 | 0.000 | 0.054 | 0.044 | 0.114 | 0.055 | 0.071 | 0.066 | **0.241** | 0.019 | - | 0.002 | 0.000 | 0.041 |
| L1 | 0.049 | 0.034 | 0.00 | 0.000 | 0.007 | 0.000 | 0.000 | 0.000 | 0.000 | 0.000 | 0.000 | 0.000 | 0.000 | 0.000 | 0.000 | 0.000 | - | **0.265** | **0.469** |
| L2 | 0.000 | 0.000 | 0.000 | -0.01 | 0.000 | 0.000 | 0.035 | 0.000 | 0.000 | 0.000 | 0.028 | 0.075 | 0.000 | 0.007 | 0.024 | 0.000 | **0.424** | - | **0.217** |
| L3 | 0.045 | 0.075 | 0.000 | 0.000 | 0.000 | 0.000 | 0.000 | 0.000 | 0.050 | 0.017 | 0.000 | 0.000 | 0.000 | 0.039 | 0.000 | 0.000 | **0.440** | **0.282** | - |

A: Anxiety symptoms, items from the Generalized Anxiety Disorder-7 item scale; D: Depression symptoms, items from the Patient Health Questionnaire-9 item scale; L: Loneliness, items from the UCLA-3 item loneliness scale.

**Supplementary Table 4.** Summary of significant edge differences between pre-COVID-19 network and during COVID-19 network after Bonferroni-Holm correction

| Edges | Pre-COVID-19 | During COVID-19 | Absolute difference | p |
| --- | --- | --- | --- | --- |
| D1 – D3 | -0.060 | 0.000 | -0.060 | <.001 |
| D2 – D3 | 0.000 | 0.044 | 0.044 | .013 |
| D2 – D4 | -0.040 | 0.084 | 0.124 | <.001 |
| D3 – D6 | 0.015 | -0.039 | 0.054 | <.001 |
| D3 – D7 | -0.056 | 0.007 | 0.062 | <.001 |
| D4 – D7 | 0.000 | 0.075 | 0.075 | <.001 |
| D6 – D8 | 0.026 | 0.115 | 0.089 | <.001 |
| D7 – D8 | 0.000 | 0.154 | 0.154 | <.001 |
| D5 – D9 | 0.095 | 0.022 | 0.072 | .045 |
| D2 – A3 | 0.067 | 0.000 | 0.067 | .024 |
| D4 – A4 | 0.000 | 0.044 | 0.044 | <.001 |
| D8 – A5 | 0.158 | 0.097 | 0.061 | .045 |
| A1 – A6 | 0.272 | 0.193 | 0.079 | .04 |
| A1 – A7 | 0.114 | 0.028 | 0.086 | .03 |
| A3 – A7 | 0.071 | 0.162 | 0.092 | .013 |
| L1 – L2 | 0.424 | 0.265 | 0.160 | <.001 |
| D1 – L3 | 0.045 | 0.000 | 0.045 | .04 |
| D6 – L3 | 0.000 | 0.040 | 0.040 | .01 |
| A7 – L3 | 0.000 | 0.041 | 0.041 | <.001 |

A: Anxiety symptoms, items from the Generalized Anxiety Disorder-7 item scale; D: Depression symptoms, items from the Patient Health Questionnaire-9 item scale; L: Loneliness, items from the UCLA-3 item loneliness scale.

**
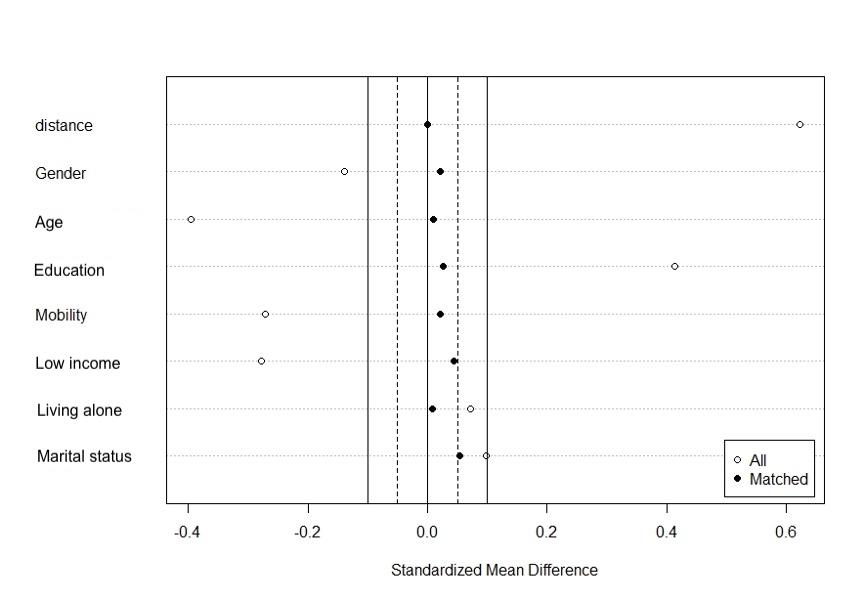
**

**Supplementary Figure 1.** Standardized bias (%) across covariates in the propensity score before and after matching.


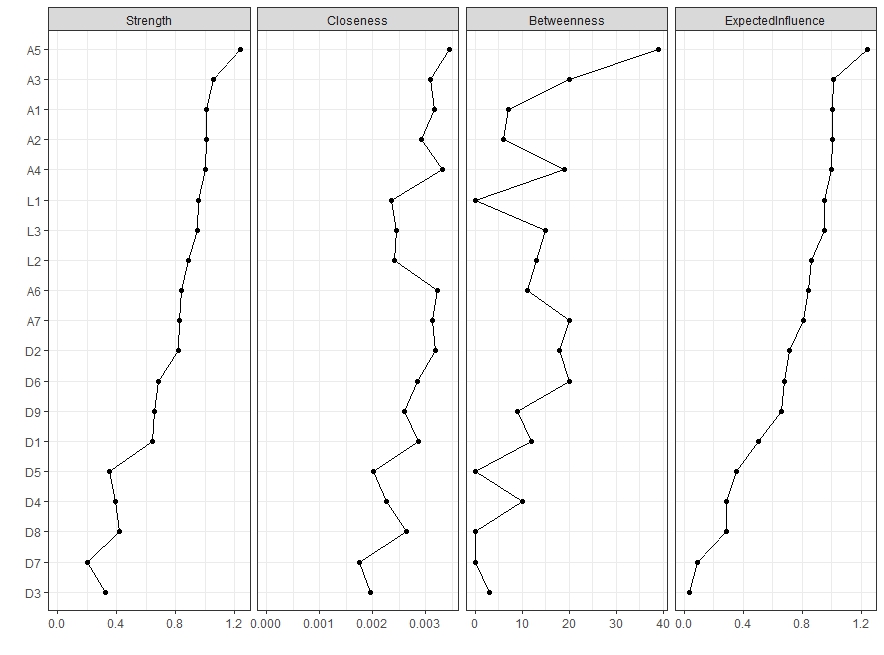

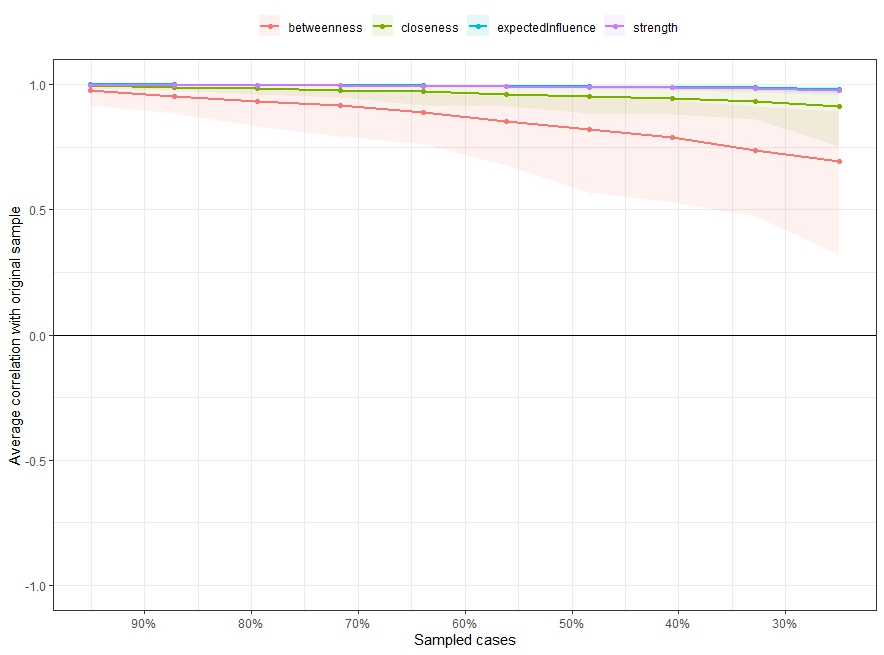


a)

b)

**Supplementary Figure 2a)** Centrality measure (Strength, Betweenness, Closeness) of all items within the network before COVID-19 (*z*-scores). **b)** Stability of centrality indices by case dropping subset bootstrap. The *x*-axis represents the percentage of cases of the original sample used at each step. The *y*-axis represents the average of correlations between the centrality indices from the original network and the centrality indices from the networks that were re-estimated after excluding increasing percentages of cases. Each line indicates the correlations among betweenness, closeness, strength, and expected influence.


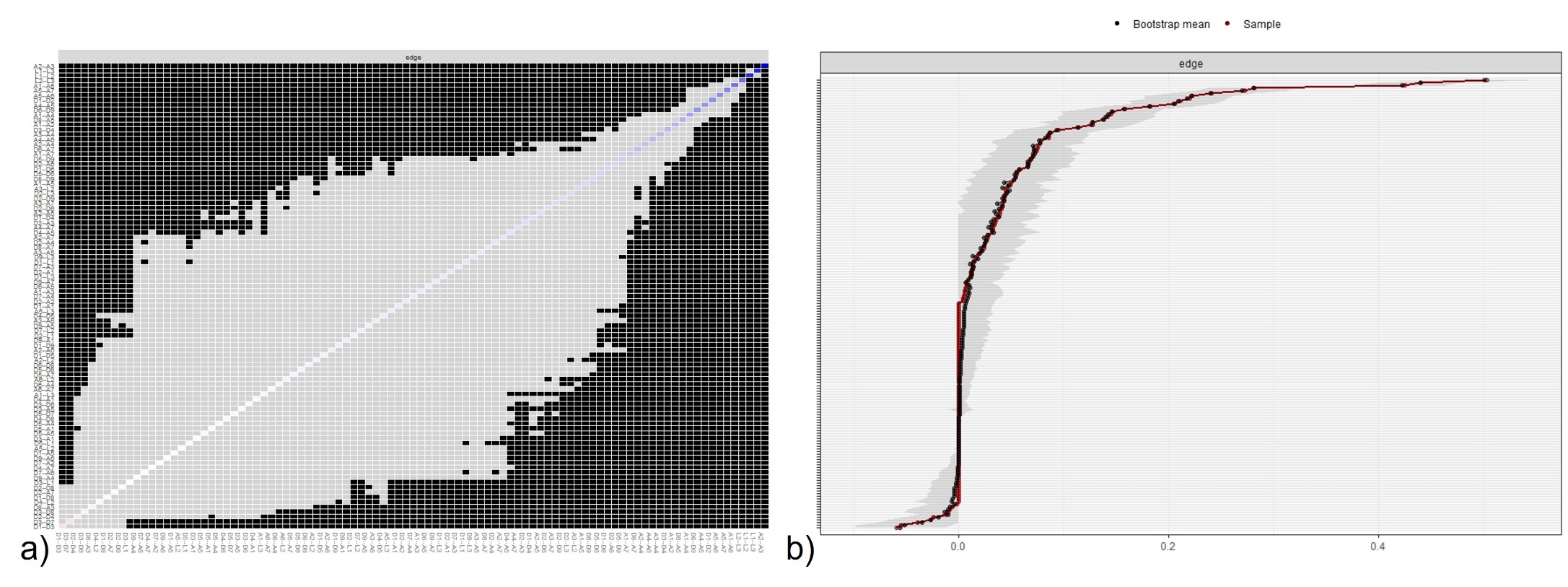


**Supplementary Figure 3a)** Nonparametric bootstrapped difference test for edges in networks of before COVID-19 sample. Gray boxes indicate no significant difference, whereas black boxes indicate a statistically significant difference (*p* < 0.05). Diagonal color and saturation represent the magnitude and direction of each estimated edge. **b)** Bootstrapped confidence intervals of edge weights in the before COVID-19 sample. The black dots indicate the values of each edge weight, ordered from the highest to the lowest value. The gray area represents the 95% Confidence

**Supplementary Figure 4a)** Centrality measure (Strength, Betweenness, Closeness) of all items within the network during COVID-19 (*z*-scores). **b)** Stability of centrality indices by case dropping subset bootstrap. The *x*-axis represents the percentage of cases of the original sample used at each step. The *y*-axis represents the average of correlations between the centrality indices from the original network and the centrality indices from the networks that were re-estimated after excluding increasing percentages of cases. Each line indicates the correlations among betweenness, closeness, strength, and expected influence.


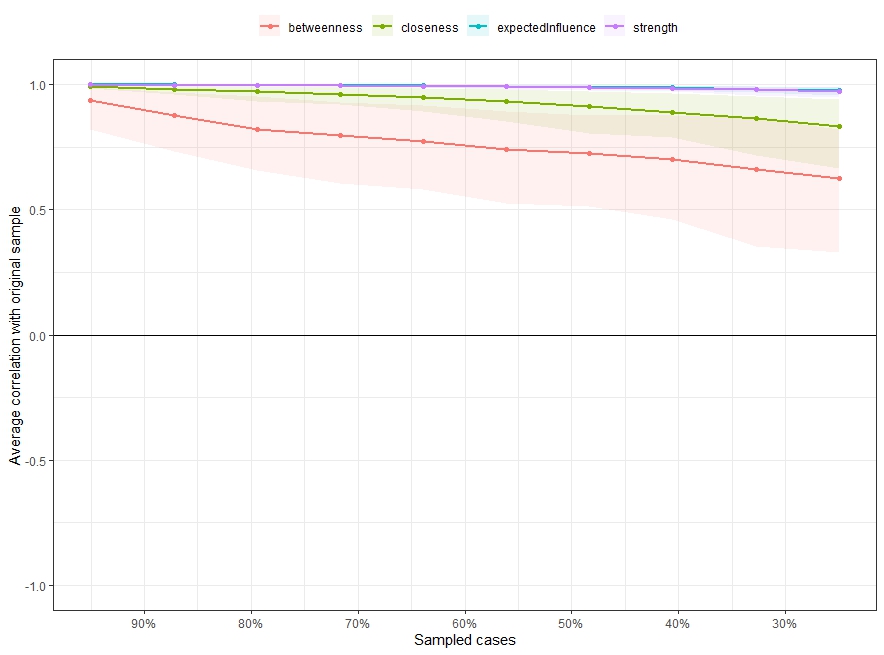

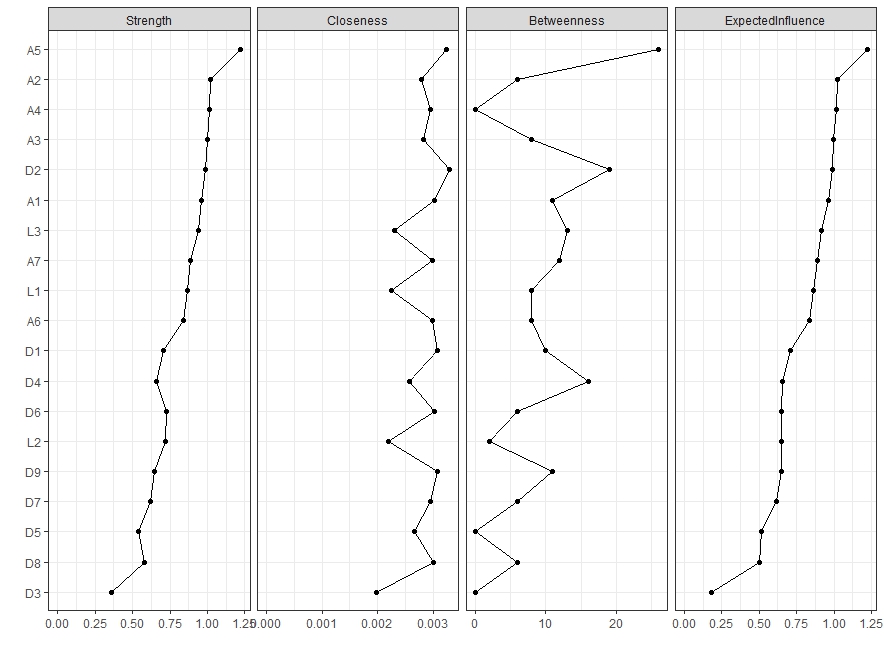


a)

b)


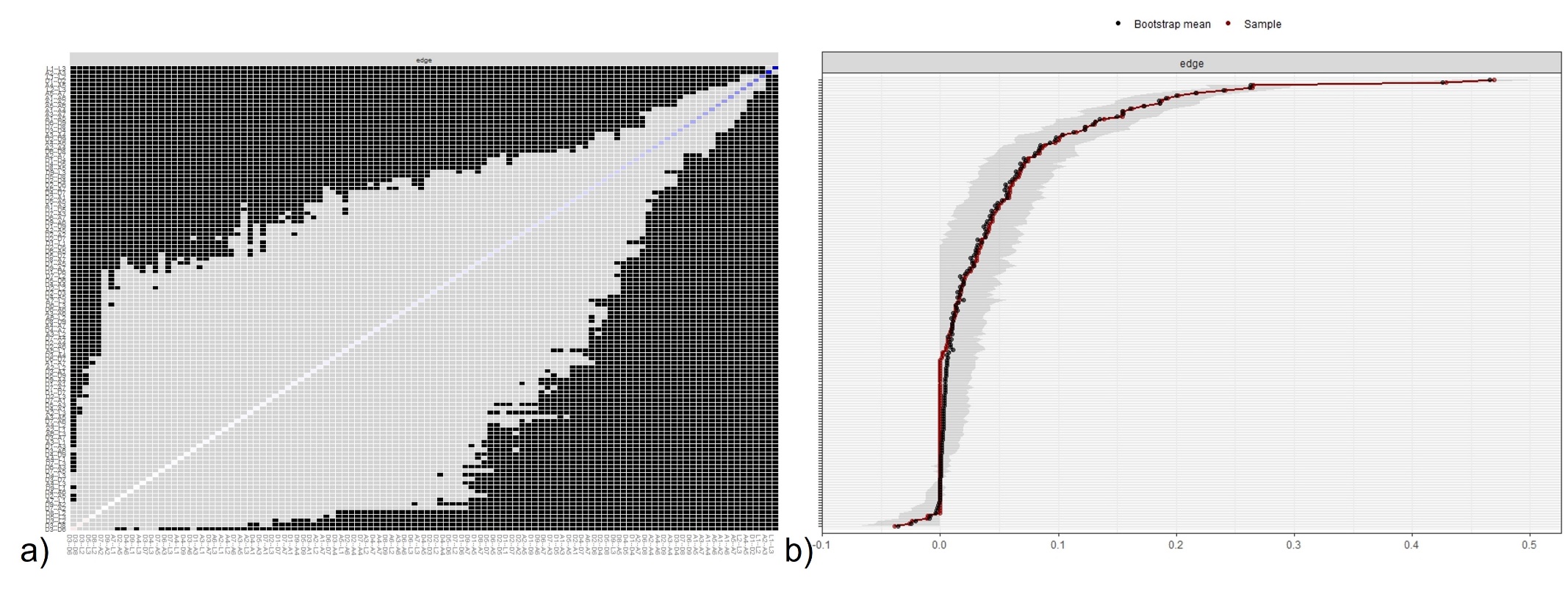


**Supplementary Figure 5a)** Nonparametric bootstrapped difference test for edges in networks of before COVID-19 sample. Gray boxes indicate no significant difference, whereas black boxes indicate a statistically significant difference (*p* < 0.05). Diagonal color and saturation represent the magnitude and direction of each estimated edge. **b)** Bootstrapped confidence intervals of edge weights in the before COVID-19 sample. The black dots indicate the values of each edge weight, ordered from the highest to the lowest value. The gray area represents the 95% Confidence Intervals of edge weights, estimated with the non-parametric bootstrap procedure. Wide intervals indicate lower stability, and narrow intervals indicate higher stability.
